# Supplementary material for: Evolution of the SARS-CoV-2 mutational spectrum
Source: bioRxiv. 2022 Nov 21:2022.11.19.517207. Preprint. [Version 1] doi: 10.1101/2022.11.19.517207 (PMC9709787; doi:10.1101/2022.11.19.517207)
Supplement: 1 [file NIHPP2022.11.19.517207V1-supplement-1.pdf]

bioRxiv preprint doi: https://doi.org/10.1101/2022.11.19.517207; this version posted November 21, 2022. The copyright holder for this preprint (which was not certified by peer review) is the author/funder, who has granted bioRxiv a license to display the preprint in perpetuity. It is made available under aCC-BY 4.0 International license.

Supplementary Material

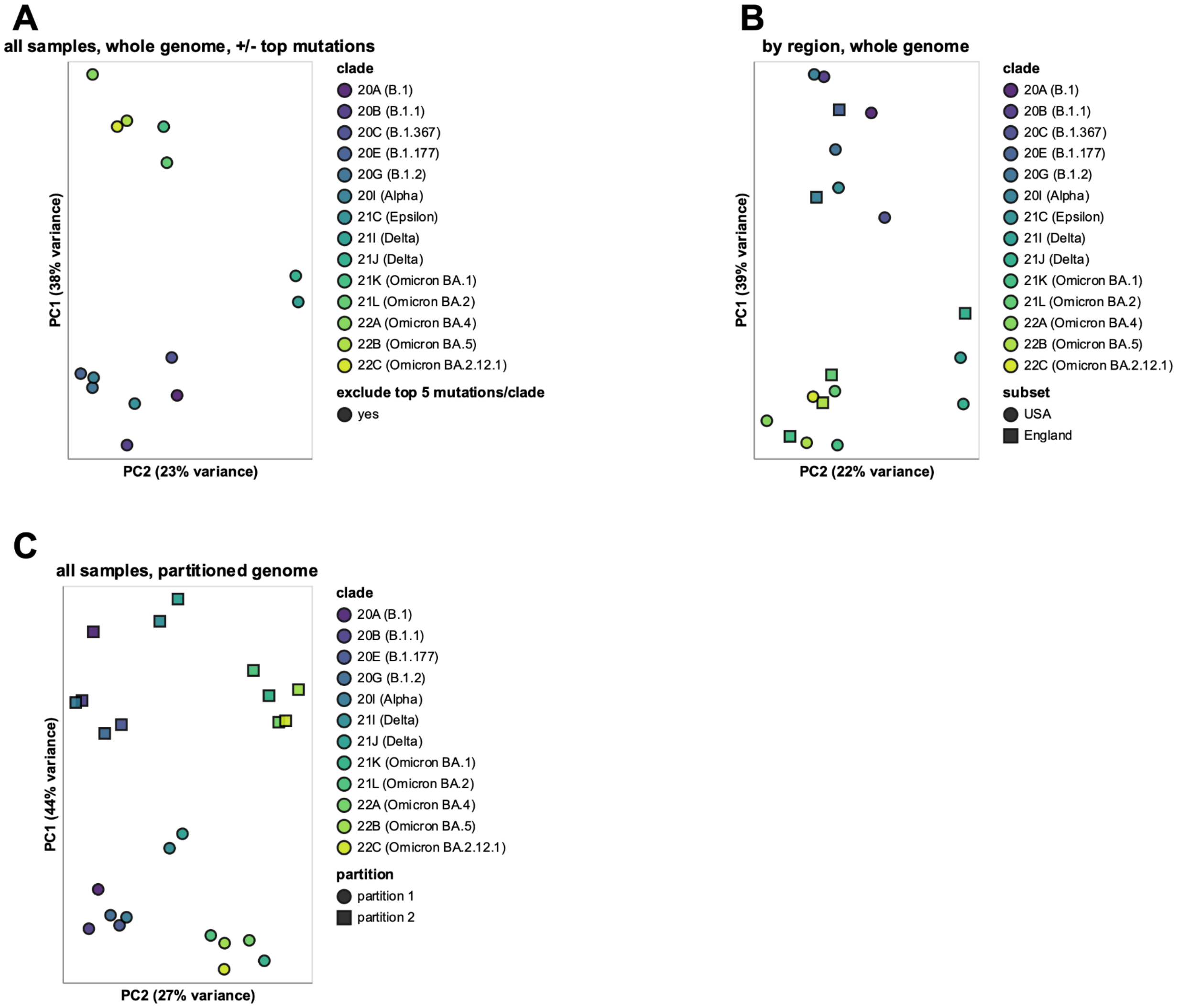

Figure S1. The inter-clade differences in mutation spectrum are robust to various possible sources of noise. This figure repeats the PCA in Figure S1 and shows that the results are robust to (A) excluding sites of the top-5 most abundant mutations in each clade, (B) examining only sequences from the USA or England, or (C) partitioning the genome into half. For panel C, some structure in the PCA plot is explainable by the genome partitioning but the shift in points caused by partitioning the genome is consistent across all clades, and so is not responsible for the inter-clade differences. These plots can be more easily explored using the interactive versions at https://jbloomlab.github.io/SARS2-mut-spectrum/ that enable mousing over of points and clicking on the legend to choose specific clades or groupings.

bioRxiv preprint doi: https://doi.org/10.1101/2022.11.19.517207; this version posted November 21, 2022. The copyright holder for this preprint (which was not certified by peer review) is the author/funder, who has granted bioRxiv a license to display the preprint in perpetuity. It is made available under aCC-BY 4.0 International license.

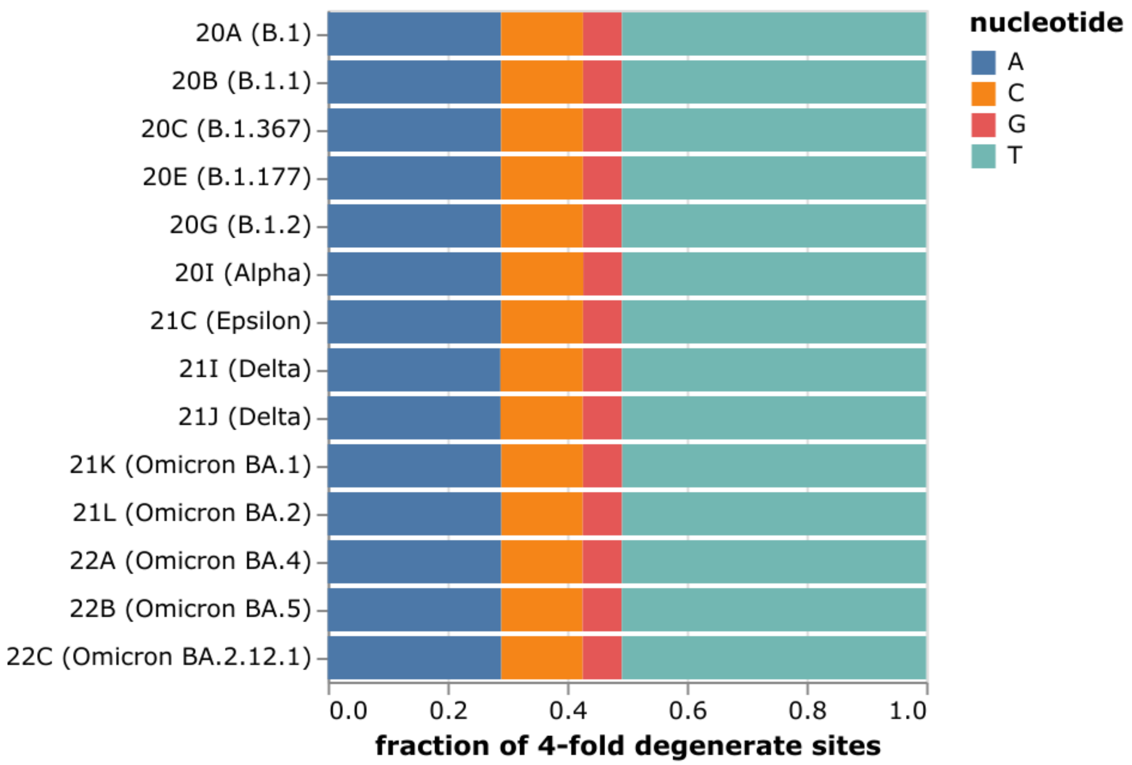

Figure S2: The frequencies of nucleotides at four-fold degenerate sites are nearly identical among the clade founder sequences.
